# The Team Is Not Okay: Violence in Emergency Departments Across Disciplines in a Health System

**DOI:** 10.5811/westjem.2022.9.57497

**Published:** 2023-02-01

**Authors:** Sarayna S. McGuire, Janet L. Finley, Bou F. Gazley, Aidan F. Mullan, Casey M. Clements

**Affiliations:** * Mayo Clinic, Department of Emergency Medicine, Rochester, Minnesota; † Mayo Clinic, Global Security, Rochester, Minnesota; ‡ Mayo Clinic, Department of Quantitative Health Sciences, Rochester, Minnesota

## Abstract

**Introduction:**

Healthcare workers, particularly those in the emergency department (ED), experience high rates of injuries caused by workplace violence (WPV).

**Objective:**

Our goal was to establish the incidence of WPV among multidisciplinary ED staff within a regional health system and assess its impact on staff victims.

**Methods:**

We conducted a survey study of all multidisciplinary ED staff at 18 Midwestern EDs encompassing a larger health system between November 18–December 31, 2020. We solicited the incidence of verbal abuse and physical assault experienced and witnessed by respondents over the prior six months, as well as its impact on staff.

**Results:**

We included responses from 814 staff (24.5% response rate) for final analysis with 585 (71.9%) indicating some form of violence experienced in the preceding six months. A total of 582 (71.5%) respondents indicated experiencing verbal abuse, and 251 (30.8%) indicated experiencing some form of physical assault. All disciplines experienced some type of verbal abuse and nearly all experienced some type of physical assault. One hundred thirty-five (21.9%) respondents indicated that being the victim of WPV has affected their ability to perform their job, and nearly half (47.6%) indicated it has changed the way they interact with or perceive patients. Additionally, 132 (21.3%) indicated experiencing symptoms of post-traumatic stress, and 18.5% reported they have considered leaving their position due to an incident.

**Conclusion:**

Emergency department staff suffer violence at a high rate, and there is no discipline that is spared. As health systems seek to prioritize staff safety in violence-prone areas such as the ED, it is imperative to recognize that the entire multidisciplinary team is impacted and requires targeted efforts for improvement in safety.

## INTRODUCTION

Healthcare workers experience high rates of injuries caused by workplace violence (WPV). Within the United States, they are five times as likely to suffer an injury as a result of violence in the workplace than workers overall in all industries.^1^ Emergency departments (ED) represent a healthcare setting where violence is commonly experienced.^2^–^12^ Prior studies have sought to establish the incidence of WPV among individual staff groups, such as clinicians^2^,^3^ and nursing staff;^4^,^13^,^14^ however, researchers seeking to establish the incidence of violence among multidisciplinary ED team members have done so at individual hospital facilities^5^,^6^ or have been limited in ancillary staff surveyed.^8^–^10^ To our knowledge, a comprehensive multidisciplinary incidence of WPV against ED staff has not been established within a large, diverse health system. Our objective in this study was to establish the incidence of WPV among multidisciplinary ED staff within a regional health system in the Midwestern US and assess its impact on staff victims.

## Methods

### Study Design and Setting

This descriptive, cross-sectional study, which took place November 18–December 31, 2020, included 18 Midwestern EDs encompassing a larger regional health system across Minnesota and Wisconsin. Survey sites included EDs in four larger, regional hospitals, four midsize hospitals, and 10 critical access hospitals with individual annual average 2019 ED patient volumes of 43,910, 15,877 and 7,703, respectively.^15^

### Security Features of Emergency Department Sites

Eight (44.4%) study sites feature a locked unit within the ED, three (16.7%) use hand-held metal detectors, and six (33.3%) report some degree of weapons screening, often passive screening, while having behavioral health patients change out of street clothes. Seven (38.9%) of the sites indicated 24/7 hospital security staffing, one (5.6%) indicated staffing seven nights a week (6 pm – 6:30 am), one (5.6%) reported staffing five days a week, seven (38.9%) reported staffing three days a week, and two (11.1%) indicated no scheduled security staffing. Among the 16 sites with security staffing, one (6.3%) had 24/7 security staffing within the ED, two (12.5%) had part-time dedicated ED security staff, and the remainder of sites (13; 81.3%) indicated no dedicated ED security staffing. The site with 24/7 ED security availability also implemented a part-time police officer program (2 pm – 2 am) with three local law enforcement officers during the study period. Police officers at this site served as a law enforcement service within the hospital and a resource to staff but did not perform a security role within the department.

### Selection of Participants

The target population consisted of all multidisciplinary staff who work within the ED, including non-ED staff assigned to other departments that perform services for ED patients. This population included clinicians (attending and resident physicians as well as advanced practice providers), nursing staff and patient care assistants, unit secretaries, ancillary testing service personnel (electrocardiogram, urology [responsible for placing all indwelling urinary catheters at one site), radiology, and phlebotomy)], registration/finance staff, paramedics/emergency medical technicians (EMT) (responsible for providing clinical assistance at some sites), social workers, respiratory therapists, housekeeping staff, and security officers. After institutional review board (IRB) review, the survey was distributed broadly by department and job type to anyone who might work in the ED even occasionally, via email distribution lists to the target population with a cover letter describing the study purpose, directions for participation, and information regarding informed consent.

**Population Health Research Capsule**

What do we already know about this issue?

*Healthcare workers, particularly those in the ED, experience high rates of injuries caused by workplace violence (WPV)*.

What was the research question?

*What is the incidence and impact of WPV among multidisciplinary ED staff within a regional health system?*

What was the major finding of the study?

*ED staff suffer violence at a high rate (71.9%) and no discipline is spared. Nearly half reported changing how they interact with patients, 21.3% reported post-traumatic stress, and 18.5% considered leaving their position*.

How does this improve population health?

*As health systems seek to prioritize staff safety in violence-prone areas, it is imperative to recognize that the entire multidisciplinary team is impacted*.

The survey was sent electronically to 3,397 staff members, although these distribution lists also included some hospital staff not working in the ED, who would not participate as the scope of the questions was limited to ED work. The questionnaire included a statement of informed consent at the beginning, and completion indicated participant consent for inclusion in the study. Three reminder notices were sent through the same method prior to the close of the survey. The IRB reviewed this study and materials and deemed it exempt from approval requirement.

### Measurements

We developed an anonymous online survey (Qualtrics LLC, Provo, UT) that included single-choice, multiple-choice. and Likert-scale response questions. This survey was based on and expanded from a previous survey developed and used in McGuire et al.^6^ Participants were asked to indicate whether they had experienced any verbal abuse or physical assault in the prior six months (May/June–November/December 2020) while working in the ED. If answering affirmatively, respondents were directed by survey branching logic to indicate what type of abuse/assault they had experienced, who was the offender (patient, visitor, or coworker), and whether they had reported the incident.^16^ Participants were also surveyed on verbal abuse and physical assault witnessed against coworkers with similar branching logic.

We used Likert scales to measure participants’ perception of safety and estimated frequency of verbal abuse and physical assault. Study participants were also asked a series of questions to assess the impact that WPV has had on them, including whether it has impacted their ability to perform their job, whether they have taken time off from work or considered leaving their position, whether it has changed the way they interact with or perceive patients, or whether they have experienced any signs or symptoms of post-traumatic stress (flashbacks, severe anxiety, emotional numbing, diminished interest in everyday activities, or detachment from others) as a result of an incident of WPV.^17^ We collected standard demographic measures. To encourage survey completion, questions were made optional for respondents to complete.

### Outcomes

The primary outcome was the incidence of verbal abuse and physical assault experienced and witnessed by multidisciplinary ED staff in a six-month time frame as indicated by survey responses. The secondary outcome was the reported impact of this violence on staff.

### Data Analysis

We summarized survey responses with frequency counts and percentages. Subgroup comparisons of survey responses were made using chi-squared tests. We compared the frequency of violence experienced from patients, visitors, and colleagues using relative risk ratios (RR) with 95% confidence intervals.

## RESULTS

A total of 833 respondents completed the survey. We excluded the responses of 19 participants who indicated primary employment at two sites not included in the study cohort because those sites were not fully integrated within the health system. Fourteen respondents indicated working primarily in a management position. As these responses came directly from the targeted distribution lists and may have included some patient care responsibilities in addition to their managerial role, they were included among the 814 total responses used for final analysis. Cohort demographics are provided in Table 1.

### Incidence of Workplace Violence

Overall, 585 (71.9%) respondents indicated experiencing some form of violence in the preceding six months, and 545 (67.0%) indicated witnessing a form of violence directed against a coworker. Further, 582 respondents (71.5%) indicated experiencing verbal abuse, and 537 (66.0%) indicating observing verbal abuse directed against a coworker (Table 2). Two hundred fifty-one (30.8%) respondents indicated experiencing some form of physical assault in the preceding six months, and 286 (35.1%) indicated witnessing a form of physical assault directed against a coworker.

Reported frequency of verbal abuse from patients or visitors (N=720) included every day or two (50; 6.9%); every week (110; 15.3%); every month (166; 23.1%); less than once a month (156; 21.7%); and 1–2 times a year (140; 19.4%), while 98 respondents (13.6%) indicated they had never experienced verbal abuse. Reported frequency of physical assault inflicted by patients or visitors (719) included every day or two (1; 0.1%); every week (15; 2.1%); every month (43; 6.0%); less than once a month (103; 14.3%); and 1–2 times a year (180; 25.0%), with 377 respondents (52.4%) indicating never experiencing physical assault.

When comparing survey site groupings (regional hospitals, midsize hospitals, and critical access hospitals), we found no statistical difference in the overall incidence of violence or incidence of verbal abuse between groups; however, the incidence of physical assault was lower at critical access hospitals (19/121; 15.7%), compared to midsize hospitals (36/102; 35.3%; *P*=.001) and regional hospitals (173/450; 38.4%; *P*<.001).

Nursing staff, clinicians, and security personnel experienced the highest rates of verbal abuse, with over 91% of respondents in these roles reporting some form of verbal abuse (Table 3). Security personnel were more likely to receive personal threats compared to nursing staff or clinicians (68.1% vs 44.2%, *P*=.004). Housekeeping and ED management staff were the least likely to experience verbal abuse, with 42.9% of ED management and 8.3% of housekeeping staff experiencing some form of verbal abuse. These positions experienced significantly less verbal abuse compared to all other positions (18.0% vs 67.7%, *P*<.001). There was no significant difference in harassment personally experienced by respondents based on race (15.2% White respondents vs 14.8% non-White respondents, *P*>.99).

Nursing staff, clinicians, and security personnel also experienced the highest rates of physical assault (Table 3). Security personnel had the highest rate at 78.7%, which was significantly higher than clinicians and nursing staff (78.7% vs 47.3%, *P*<.001) as well as all non-security positions (78.7% vs 25.2%, *P*<.001). Housekeeping staff, social workers, and unit secretaries had the lowest rates of physical assault, with less than 9% of respondents from these job positions indicating any form of physical violence. Staff working ≥6 months in their ED were more likely to have experienced any type of verbal abuse (*P*<.001) and physical violence (*P*<.001) compared to those working <6 months in their ED.

### Perpetrators of Violence

Among the 766 respondents who provided data, 545 (71.1%) indicated experiencing verbal abuse from patients, 223 (29.1%) from visitors, and 66 (8.6%) from coworkers. The risk of verbal abuse was nearly 2.5 times greater from patients than visitors (RR 2.44, 95% CI 2.17–2.75; *P*<.001) and over eight times greater from patients than coworkers (RR 8.26, 95% CI 6.53–10.45; *P*<.001). The risk of experiencing verbal abuse from visitors was 3.4 times greater than the risk of verbal abuse from coworkers (RR 3.38, 95% CI 2.62–4.36, *P* <.001).

Physical assault was most commonly perpetrated by patients, with 248 (32.7%) of 759 respondents indicating some form of physical assault from patients. The risk of assault from patients was 62 times greater than the risk of assault from visitors (4/759 respondents, 0.5%; RR 62.0, 95% CI 23.2–165.6; *P*<.001) and over 100 times greater than the risk of assault from coworkers (2/759 respondents, 0.3%; RR 124.0, 95% CI 31.0–496.8; *P*<.001).

### Employee Impact of Violence

One-hundred and thirty-five (21.9%) respondents indicated that being the victim of WPV has affected their ability to perform their job (Table 4). The time duration of this impact included one shift or day (63, 47.0%); 2–7 days (39, 29.1%); and ≥2 weeks (32, 23.9%), with 17 (12.7%) of these respondents indicating their work was affected for ≥5 months. Nearly half of respondents (293, 47.6%) indicated that being the victim of WPV had changed the way they interact with or perceive patients. One-hundred and thirty-two (21.3%) indicated experiencing symptoms of post-traumatic stress as a result of an incident of WPV, and 127 (18.5%) reported they have considered leaving their position due to an incident.

## DISCUSSION

Similar to findings from an earlier survey study specific to a single academic institution (regional hospital),^6^ we found a high incidence of verbal abuse (71.5%) and physical assault (30.8%) directed toward multidisciplinary staff in EDs across this Midwest health system. Despite the academic department being the only site to have 24/7 dedicated ED security presence, our prior research demonstrated a higher incidence of verbal abuse (86%) and physical assault (37%) within our academic ED, compared to the larger health system cohort.^8^ This finding is contrary to prior literature that documented a higher rate of violent crime against ED staff in smaller hospitals.^12^ This is likely not explained by the timing of surveys with the COVID-19 pandemic, as we have also previously shown a positive association between the monthly hospital referral region COVID-19 case rate and rate of violent ED incidents, as well as an increase in violent incidents overall during the pandemic, and this study was sent out during an active wave of the pandemic within our region.^7^ It is more likely that this difference can be accounted for by prior methodology, with the exclusion of new hires (those working <6 months in the ED) with our first study and the lack of their exclusion in this study. This is made even more evident when, in this current study, we demonstrated that staff working ≥6 months in their ED were more likely to have experienced violence compared to those working <6 months. This finding is similar to prior literature that has demonstrated more experienced ED staff feel less safe.^9^

Contrary to prior literature that documented a higher rate of violent crime against ED staff in smaller hospitals,^12^ we found no statistical difference in the overall incidence of violence or verbal abuse between survey-site groupings (regional hospitals, midsize hospitals, and critical access hospitals); however, we did find that smaller, critical access sites had a significantly lower incidence of physical assault during the study period. Future research should attempt to identify the reason(s) for this difference in physical assault between sites.

Alarmingly, all staff disciplines experienced some type of verbal abuse and nearly all, except for housekeeping, experienced physical assault within the study period. Our study demonstrates that certain disciplines fall into different risk categories. High-risk positions for verbal abuse include clinicians, nursing, and security; medium-risk positions include respiratory therapists, social workers, paramedics/EMTs, unit secretaries, and registration/finance clerks; and lower risk positions include ancillary testing services, housekeeping, and management. High-risk positions for physical assault remain the same (clinicians, nursing, and security), whereas medium-risk positions include ancillary testing services, respiratory therapy, management, and paramedics/EMTs. Lower risk positions for physical assault include registration/finance, unit secretaries, housekeeping, and social workers. That said, the level of violence suffered by even the lower risk positions was still significant and staggering, with many of these personnel still reporting abuse in the prior six months. It is imperative that institutions and the general public recognize that all multidisciplinary team members experience WPV, including disciplines that have not historically been targeted for protective strategies or “burnout campaigns.”^18^ Recognizing that all team members are impacted and that there are differing levels of risk based on discipline can help drive future institutional policies and preventative measures.

It is worth noting that violence in healthcare is not generally related to mental illness (previously reported as a cause of ED violence in only 5.4% of assaults); in fact, the majority of violence is related to chemical health (eg, intoxication, withdrawal, and drug-seeking behaviors) (>70%).^14^ Additionally, while we found a significant amount of verbal abuse from family/visitors, it is interesting to note that physical violence was overwhelmingly committed by patients and not visitors. This distinction deserves additional attention and study as this key difference may reveal heretofore unknown prevention strategies as it relates to patients. Further details on patient characteristics or care episode characteristics (eg, length of stay, boarding, medication use, wait times) were not available based on the survey nature of the data. Future study is needed to better determine additional patient/care factors associated with violence.

A small but not insignificant amount of verbal abuse and physical assault was reported to have come from coworkers. We strongly advocate for increased reporting among staff of all violent incidents, verbal abuse, harassment, and microaggressions, regardless of perpetrator or clinical setting, in accordance with the premise that apathy toward low-level events creates an environment conducive to more serious offenses.^11^ The need for zero tolerance for violence in healthcare is made even more evident by our findings that 1 in 5 of our cohort felt that being the victim of workplace violence had affected their ability to perform their job and nearly 1 in 2 felt it had changed the way they interacted with or perceived patients. Concerningly, 1 in 5 reported symptoms of post-traumatic stress due to workplace violence. Similar to prior literature, we found that a significant number of staff within our cohort have considered leaving their job as a result of violence.^3^,^13^

## LIMITATIONS

This study has several important limitations. To preserve anonymity of employees, the study was sent to email distribution lists and included some lists with employees who worked in other departments other than the ED (eg, phlebotomy, ECG, and radiology technicians). Thus, it was not possible to determine the actual number of employees from different disciplines who work in their respective EDs, and we could only estimate a response rate for this survey study. We also recognize the potential for nonresponse bias in that respondents who had not experienced WPV may not have completed the survey. Certainly, we would anticipate that victims of traumatic events may be more or less likely to respond to a survey in which they would be asked to recount details of those events. Additionally, we could not control for a true nonresponse rate due to the use of email distribution lists, where individuals on those lists who did not work in the ED during the study period were instructed not to respond to the survey.

Given that the definition of “verbal abuse” is highly subjective, survey inclusion of “threatening tone of voice” may have contributed to over-reporting of verbal abuse in general by respondents. The study was also subject to recall and reporting bias in terms of recalling violence experienced or reporting incidents over a six-month period. Although this was a multicenter study, it was localized to a specific health system and region within the United States; therefore, some aspects may not be generalizable to all institutions or geographic regions. However, the findings of significant incidence of verbal abuse and physical assault experienced by ED staff are not dissimilar to other published studies. Our findings that abuse and violence affect previously unstudied populations, including ancillary services and support staff, is important and not likely related to local factors.

## CONCLUSION

We found a high incidence of verbal abuse (71.5%) and physical assault (30.8%) directed toward multidisciplinary staff in EDs across our Midwest health system. All staff disciplines experienced some type of verbal abuse, and nearly all experienced physical assault within the study period. Alarmingly, 1 in 5 of our cohort felt that being the victim of workplace violence affected their ability to perform their job and nearly 1 in 2 agreed it had changed the way they interact with patients; 1 in 5 reported symptoms of post-traumatic stress, and nearly 1 in 5 reported that they had considered leaving their job as a result of a violent incident.

## Figures and Tables

**Table 1:** Respondent demographics.†

	N (%)
Gender (N = 658)	
Male	172 (26.1%)
Female	483 (73.4%)
Transgender	3 (0.5%)
Race (N = 814)	
White	638 (78.4%)
Non-White	176 (21.6%)
Ethnicity (N = 661)	
Hispanic/Latino	19 (2.9%)
Not Hispanic/Latino	642 (97.1%)
Worked in ED for 6 months (N = 814)	
Yes	728 (89.4%)
Primary role in ED (N = 683)	
Clinicians	109 (16.0%)
Nursing staff	208 (30.5%)
Testing services	119 (17.4%)
Social work	28 (4.1%)
Housekeeping	36 (5.3%)
Paramedic/EMT	12 (1.8%)
Unit secretary	12 (1.8%)
Registration/finance	75 (11.0%)
Security	47 (6.9%)
Management	14 (2.0%)
Respiratory therapy	23 (3.4%)
Employment status (N = 678)	
Full time	364 (53.7%)
Part time	286 (42.4%)
Supplemental^1^	28 (4.1%)
Primary shift (N = 680)	
Day	255 (37.5%)
Evening	80 (11.8%)
Night	104 (15.3%)
Rotating	241 (35.4%)
Years of experience (N = 683)	
0–4 years	190 (27.8%)
5–10 years	194 (28.4%)
11–20 years	178 (26.1%)
21+ years	121 (17.7%)
Primary ED location (N = 673)	
Regional hospital	450 (66.9%)
Midsize hospital	102 (15.2%)
Critical access hospital	121 (18.0%)

† Some questions were not fully completed, in which case the number of provided responses to each question are provided. Percentages are relative to the total number of available responses.

1 Supplemental staff are trained and credentialed ED staff brought in “as needed” for coverage without specific time commitments within the department.

*ED*, emergency department; *EMT*, emergency medical technicians.

**Table 2:** Incidence of verbal abuse and physical assault over the prior six months.†

	Personal experience N (%)	Witnessed against coworkers N(%)
Verbal abuse	582 (71.5%)	537 (66.0%)
Threatening tone of voice	N = 763	N = 737
Any source	567 (74.3%)	522 (70.8%)
From patient	510 (89.9%)	488 (93.5%)
From visitor	202 (35.6%)	148 (28.4%)
From coworker	50 (8.8%)	35 (6.7%)
Reported incident	96 (16.9%)	80 (15.3%)
Abusive language	N = 758	N = 733
Any source	538 (71.0%)	494 (67.4%)
From patient	501 (93.1%)	470 (95.1%)
From visitor	168 (31.2%)	134 (27.1%)
From coworker	36 (6.7%)	23 (4.7%)
Reported incident	103 (19.1%)	77 (15.6%)
Racial harassment	N = 741	N = 712
Any source	112 (15.1%)	166 (23.3%)
From patient	96 (85.7%)	159 (95.8%)
From visitor	25 (22.3%)	30 (18.1%)
From coworker	7 (6.3%)	8 (4.8%)
Reported incident	23 (20.5%)	30 (18.1%)
Gender harassment	N = 741	N = 712
Any source	136 (18.4%)	179 (25.1%)
From patient	124 (91.2%)	171 (95.5%)
From visitor	31 (22.8%)	38 (21.2%)
From coworker	8 (5.9%)	6 (3.4%)
Reported incident	18 (13.2%)	30 (16.8%)
Sexual harassment	N = 740	N = 708
Any source	138 (18.6%)	138 (19.5%)
From patient	121 (87.7%)	130 (94.2%)
From visitor	17 (12.3%)	23 (16.7%)
From coworker	11 (8.0%)	6 (4.3%)
Reported incident	24 (17.4%)	30 (21.7%)
Threats of violence	N = 744	N = 723
Any source	232 (31.2%)	255 (35.3%)
From patient	222 (95.7%)	251 (98.4%)
From visitor	40 (17.2%)	43 (16.9%)
From coworker	2 (0.9%)	1 (0.4%)
Reported incident	65 (28.0%)	58 (22.7%)
Physical assault	251 (30.8%)	286 (35.1%)
Assault with weapons	N = 758	N = 661
Any source	17 (2.2%)	51 (7.7%)
From patient	17 (100%)	51 (100%)
From visitor	1 (5.9%)	8 (15.7%)
From coworker	0 (0%)	0 (0%)
Reported incident	8 (47.1%)	20 (39.2%)
Assault with bodily fluids	N = 756	N = 655
Any source	114 (15.1%)	186 (28.4%)
From patient	113 (99.1%)	186 (100%)
From visitor	3 (2.6%)	13 (7.0%)
From coworker	0 (0%)	0 (0%)
Reported incident	43 (37.7%)	49 (26.3%)
Physical assault (punching, biting, scratching…)	N = 757	N = 660
Any source	217 (28.9%)	266 (40.3%)
From patient	217 (100%)	265 (99.6%)
From visitor	0 (0%)	15 (5.6%)
From coworker	0 (0%)	0 (0%)
Reported incident	95 (43.8%)	79 (29.7%)
Sexual assault	N = 749	N = 654
Any source	7 (0.9%)	13 (1.9%)
From patient	5 (71.4%)	12 (92.3%)
From visitor	0 (0%)	3 (23.1%)
From coworker	2 (28.6%)	0 (0%)
Reported incident	1 (14.3%)	5 (38.5%)

† Participants’ answers to each question were optional. For the highest level questions (the presence of physical or verbal abuse), potential participant participation was the entire cohort. For subsequent questions, administered using branching logic, the available participants, for which the percentage possible is shown here, were of those who were administered the questions based on answering in the affirmative to the preceding, higher level question.

**Table 3:** Incidence of violence by job position.

Verbal Abuse							

Position	Any verbal abuse	Threatening tone	Abusive language	Racial harassment	Gender harassment	Sexual harassment	Personal threats
Clinicians (N = 109)	100 (91.7%)	94 (86.2%)	91 (83.5%)	12 (11.0%)	23 (21.1%)	13 (11.9%)	38 (34.9%)
Nursing (N = 208)	199 (95.7%)	196 (94.2%)	192 (92.3%)	50 (24.0%)	56 (26.9%)	65 (31.3%)	102 (49.0%)
Testing services (N = 119)	64 (53.8%)	59 (49.6%)	50 (42.0%)	7 (5.9%)	15 (12.6%)	10 (8.4%)	9 (7.6%)
Respiratory therapy (N = 23)	17 (73.9%)	15 (65.2%)	15 (65.2%)	1 (4.3%)	1 (4.3%)	1 (4.3%)	3 (13.0%)
Social work (N = 28)	21 (75.0%)	20 (71.4%)	19 (67.9%)	0 (0%)	1 (3.6%)	1 (3.6%)	3 (10.7%)
Housekeeping (N = 36)	3 (8.3%)	0 (0%)	2 (5.6%)	0 (0%)	0 (0%)	0 (0%)	1 (2.8%)
Paramedic/EMT (N = 12)	10 (83.3%)	10 (83.3%)	9 (75.0%)	1 (8.3%)	3 (25.0%)	4 (33.3%)	6 (50.0%)
Unit secretary (N = 12)	8 (66.7%)	8 (66.7%)	8 (66.7%)	1 (8.3%)	1 (8.3%)	2 (16.7%)	2 (16.7%)
Registration/finance (N = 75)	55 (73.3%)	53 (70.7%)	53 (70.7%)	8 (10.7%)	7 (9.3%)	7 (9.3%)	13 (17.3%)
Security (N = 47)	43 (91.5%)	42 (89.4%)	43 (91.5%)	23 (48.9%)	14 (29.8%)	15 (31.9%)	32 (68.1%)
Management (N = 14)	6 (42.9%)	6 (42.9%)	6 (42.9%)	0 (0%)	1 (7.1%)	0 (0%)	3 (21.4%)

Physical assault							

Position	Any physical assault	Assault-weapons	Assault-fluids	Assault-physical	Assault-sexual		

Clinicians (N = 109)	39 (35.8%)	1 (0.9%)	17 (15.6%)	31 (28.4%)	0 (0%)		
Nursing (N = 208)	111 (53.4%)	10 (4.8%)	58 (27.9%)	98 (47.1%)	3 (1.4%)		
Testing services (N = 119)	23 (19.3%)	0 (0%)	4 (3.4%)	22 (18.5%)	1 (0.8%)		
Respiratory therapy (N = 23)	4 (17.4%)	1 (4.3%)	1 (4.3%)	2 (8.7%)	0 (0%)		
Social work (N = 28)	2 (7.1%)	0 (0%)	0 (0%)	2 (7.1%)	0 (0%)		
Housekeeping (N = 36)	0 (0%)	0 (0%)	0 (0%)	0 (0%)	0 (0%)		
Paramedic/EMT (N = 12)	3 (25.0%)	0 (0%)	2 (16.7%)	3 (25.0%)	0 (0%)		
Unit secretary (N = 12)	1 (8.3%)	0 (0%)	1 (8.3%)	1 (8.3%)	0 (0%)		
Registration/finance (N = 75)	8 (10.7%)	3 (4.0%)	5 (6.7%)	1 (1.3%)	0 (0%)		
Security (N = 47)	37 (78.7%)	2 (4.3%)	18 (38.3%)	37 (78.7%)	0 (0%)		
Management (N = 14)	2 (14.3%)	0 (0%)	1 (7.1%)	2 (14.3%)	0 (0%)		

*EMT*, emergency medical technician.

**Table 4:** Employee impact of violence.†

	N (%)
How safe do you feel in the ED? (N = 805)	
Extremely safe	86 (10.7%)
Very safe	308 (38.3%)
Moderately safe	323 (40.1%)
Slightly safe	72 (8.9%)
Not safe at all	16 (2.0%)
Has being the victim of violence affected your ability to perform your job? (N = 617)	
Yes	135 (21.9%)
How long was your work affected? (N = 134)	
One shift	44 (32.8%)
One day	19 (14.2%)
2–7 days	39 (29.1%)
2–3 weeks	6 (4.5%)
1–4 months	9 (6.7%)
5+ months	17 (12.7%)
Has being the victim of violence changed the way you interact with or perceive patients? (N = 616)	
Yes	293 (47.6%)
Have you experienced any of the following due to an incident: flashbacks, anxiety, emotional numbing, diminished interest, or detachment from others? (N = 618)	
Yes	132 (21.3%)
Have you ever considered leaving your position due to incidents of violence? (N = 685)	
Yes	127 (18.5%)

† Some questions were not fully completed, in which case the number of provided responses to each question are provided. Percentages are relative to the total number of available responses.

*ED*, emergency department.
